# Supplementary material for: Loss of SIRT1 inhibits hematopoietic stem cell aging and age-dependent mixed phenotype acute leukemia
Source: Commun Biol. 2022 Apr 28;5:396. doi: 10.1038/s42003-022-03340-w (PMC9051098; doi:10.1038/s42003-022-03340-w)
Supplement: Supplementary file 2 — Supplementary Information [file 42003_2022_3340_MOESM2_ESM.pdf]

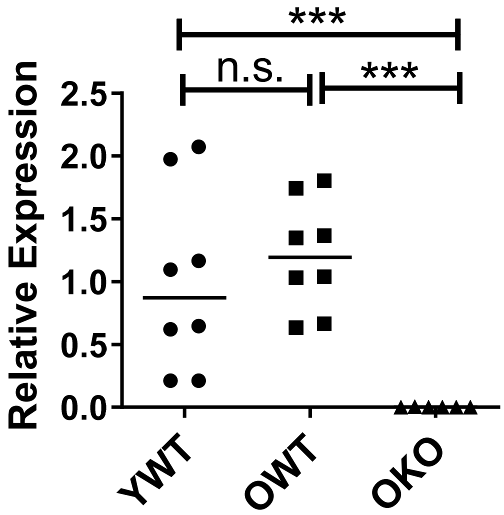

Supplementary Fig. 1 Comparison of Sirt1 mRNA expression in mouse SP HSCs by realtime RT-PCR. PCR was performed with the forward primer on the intact Sirt1 exon 4 and the reverse primer on the deleted exon 5. n.s., not significant. *** $p < 0.001$.

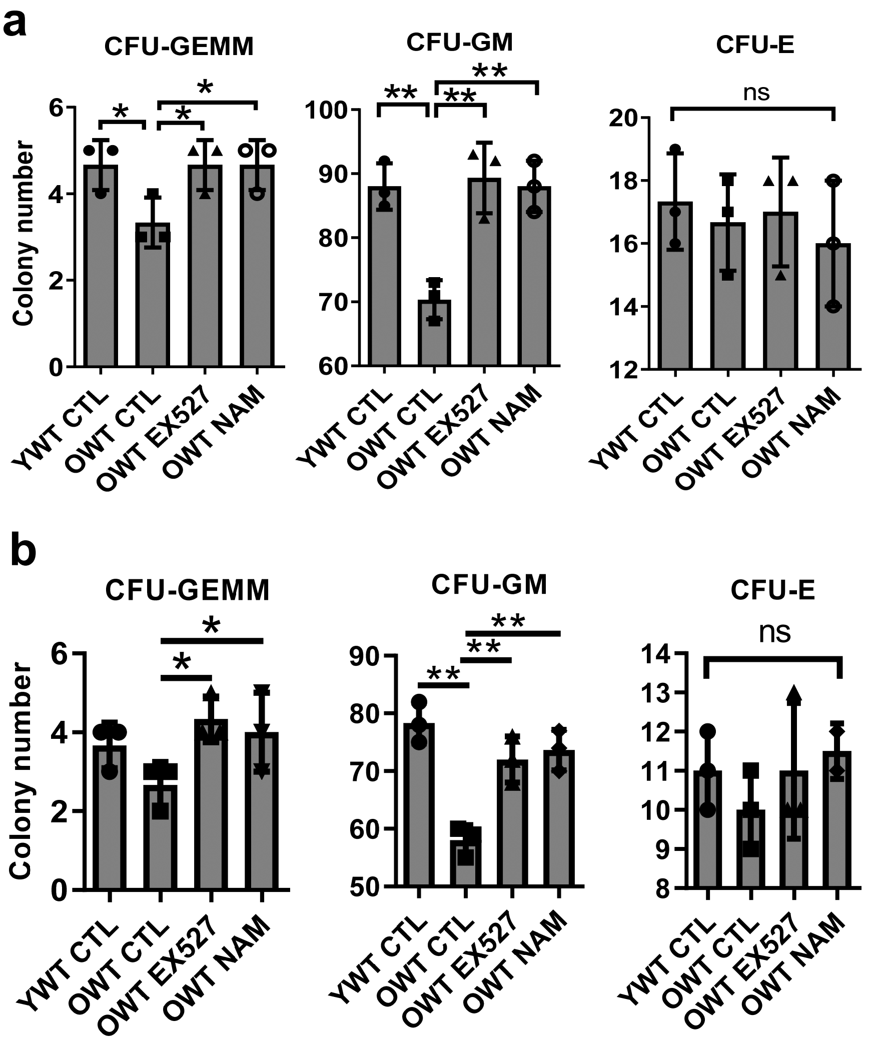

Supplementary Fig. 2 The effect of Sirt1 inhibition on aging mouse HSCs. **(A)** CFU formation of OWT bone marrow cells from 23-month-old BALB/c mice without or with treatment of a SIRT1 inhibitor, 2.5μM EX-527 or 0.2 mM nicotinamide (NAM), compared to the untreated YWT BALB/c mouse control. **(B)** CFU assay of OWT bone marrow cells from 24-month-old C57BL/6 mice. OWT cells were untreated or treated with 5μM EX-527 or 0.5 mM NAM, compared to the untreated YWT C57BL/6 mouse control. Both aging BALB/c and C57BL/6 mice were obtained from National Institute of Aging. * $p < 0.05$; ** $p < 0.01$; ns, not significant. Error bars represented one standard deviation.

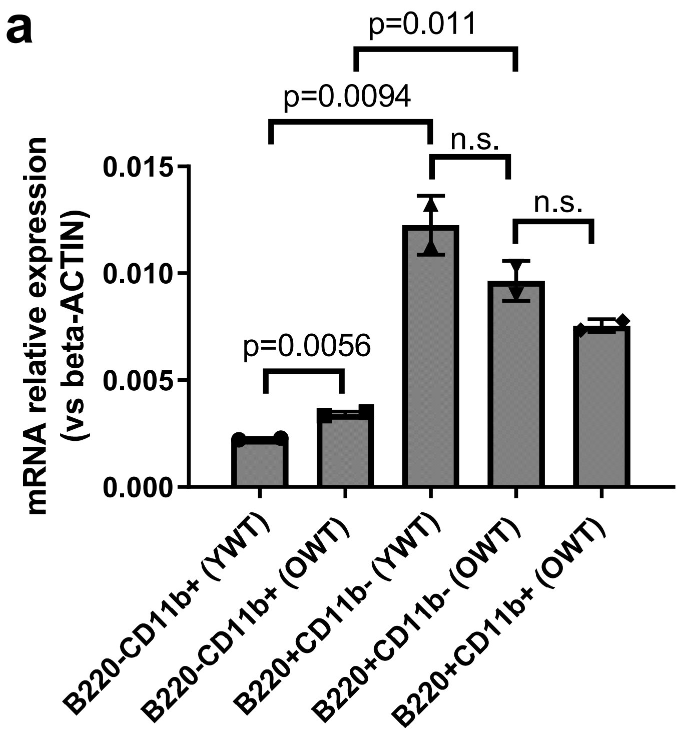

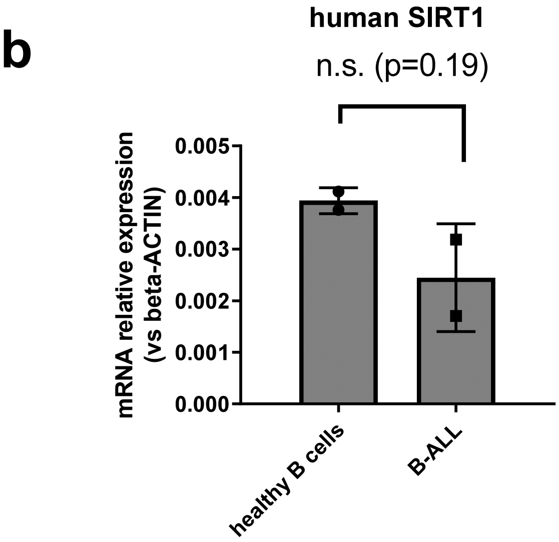

Supplementary Fig. 3 SIRT1 mRNA was not increased in MPAL and B-ALL. RT-PCR analysis of SIRT1 mRNA in normal mouse B and myeloid cells vs MPAL (A), and in CD19-enriched human adult normal B cells vs adult B-ALL blasts (B). Error bars represented one standard deviation.

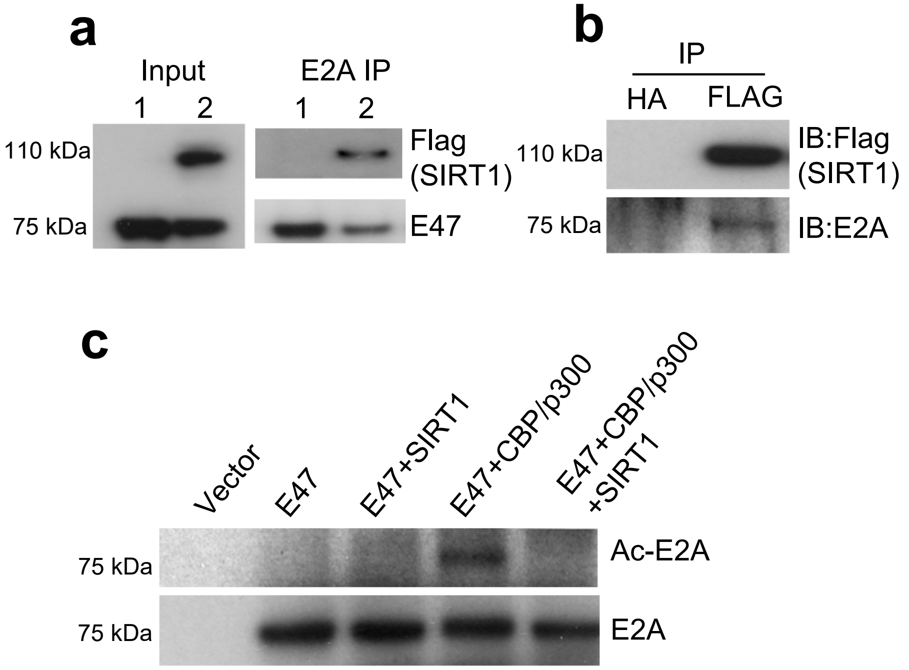

Supplementary Fig. 4 SIRT1 protein interacted with E2A protein. **(A, B)** Interaction of SIRT1 and E2A. E: E2A was co-expressed with empty vector (lane 1) or flag-SIRT1 (lane 2) in 293T cells for IP with E2A antibody, and blot was probed with an antibody for E47. F: IP with Flag (SIRT1) antibody or HA control. **(C)** SIRT1 deacetylated E2A. E47 was immunoprecipitated from 293T cells expressing the indicated constructs. The blot was probed with acetyl-lysine antibody followed by E2A antibody.

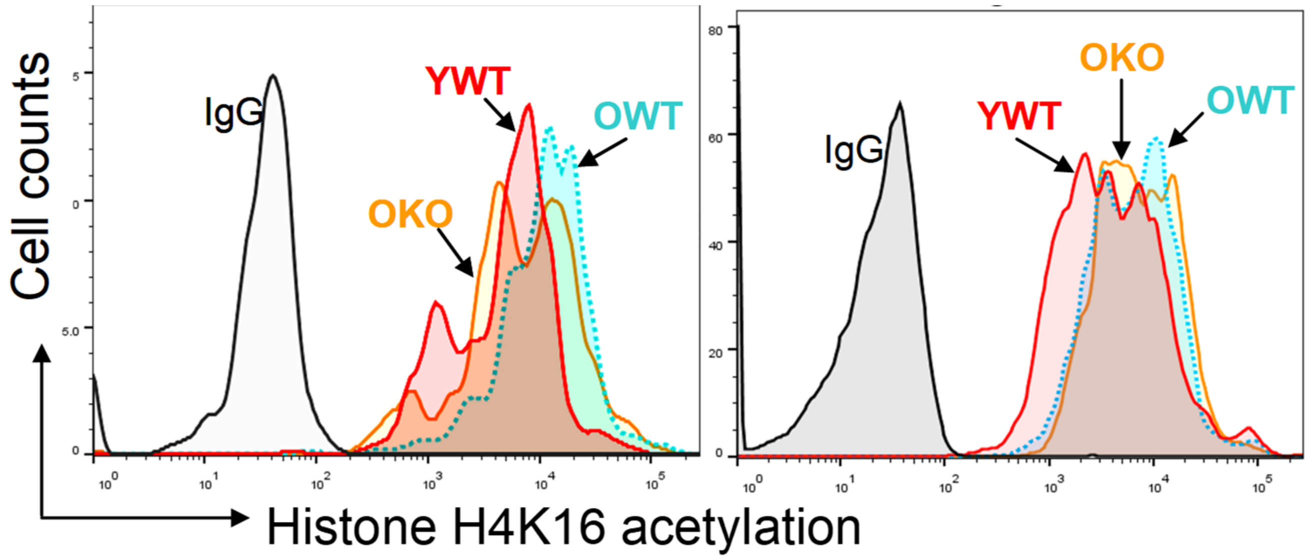

Supplementary Fig. 5 Effects of Sirt1 knockout on histone H4 K16 acetylation in aging hematopoietic stem/progenitor cells. Sirt1 knockout partially reduced H4K16Ac in less differentiated aging hematopoietic stem/progenitors enriched by $Lin^{-}CD150^{+}CD41^{-}48^{-}$ (left), but not in more differentiated cells enriched by $Lin^{-}cKit^{+}$ (right).

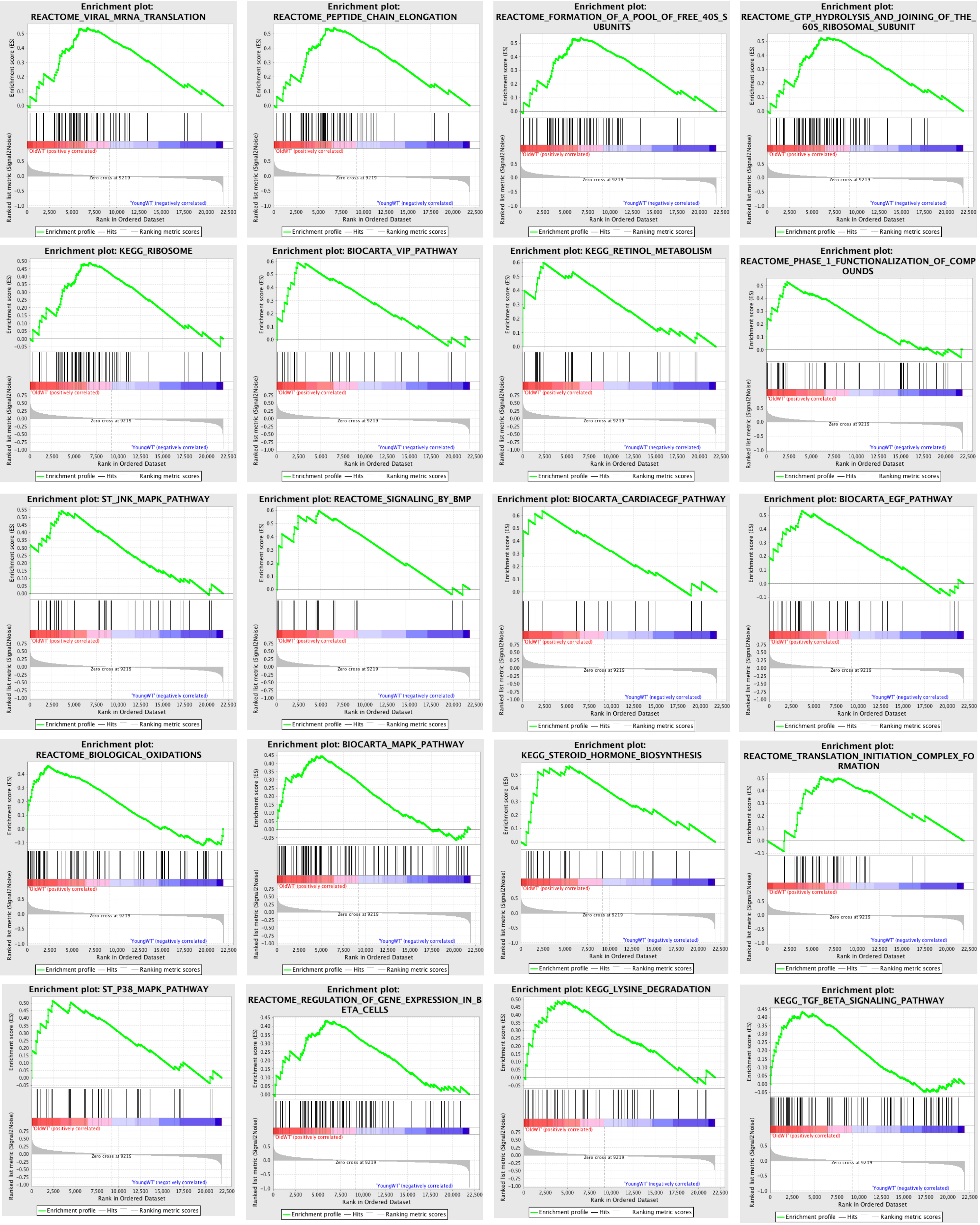

Supplementary Fig. 6 Top 20 GSEA pathways that were enriched in OWT HSCs in the analysis of OWT versus YWT HSCs. Protein synthesis pathways (viral mRNA translation, peptide chain elongation, free ribosomal 40S subunits, GTP hydrolysis and joining of ribosomal 60S subunits, KEGG ribosome, and translation initiation complex) were most prominently activated in OWT cells. GSEA plots were ranked by NES and the full list of pathways and ranking scores was provided as Supplementary Table 1.

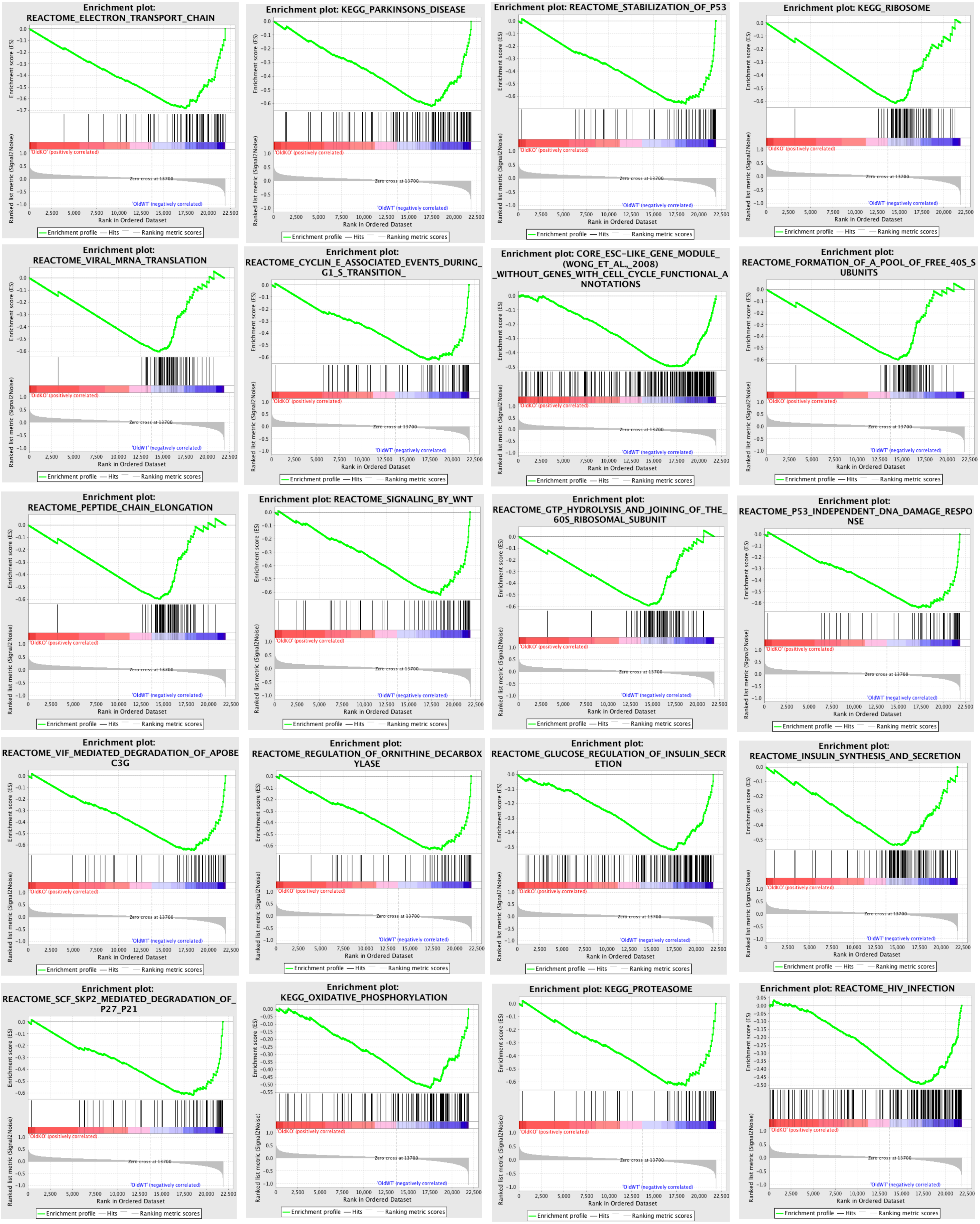

Supplementary Fig. 7 Top 20 GSEA pathways that were enriched in OWT HSCs in the analysis of OKO versus OWT HSCs. Protein synthesis pathways (KEGG ribosome, viral mRNA translation, peptide chain elongation, free ribosomal 40S subunits, GTP hydrolysis and joining of ribosomal 60S subunits) were downregulated in OKO cells along with oxidative metabolism pathways (electron transport chain and oxidative phosphorylation. Most genes in the "Parkinsons_Disease" are for the electron transport chain). GSEA plots were ranked by NES and the full list of pathways and ranking scores was provided as Supplementary Table 2.

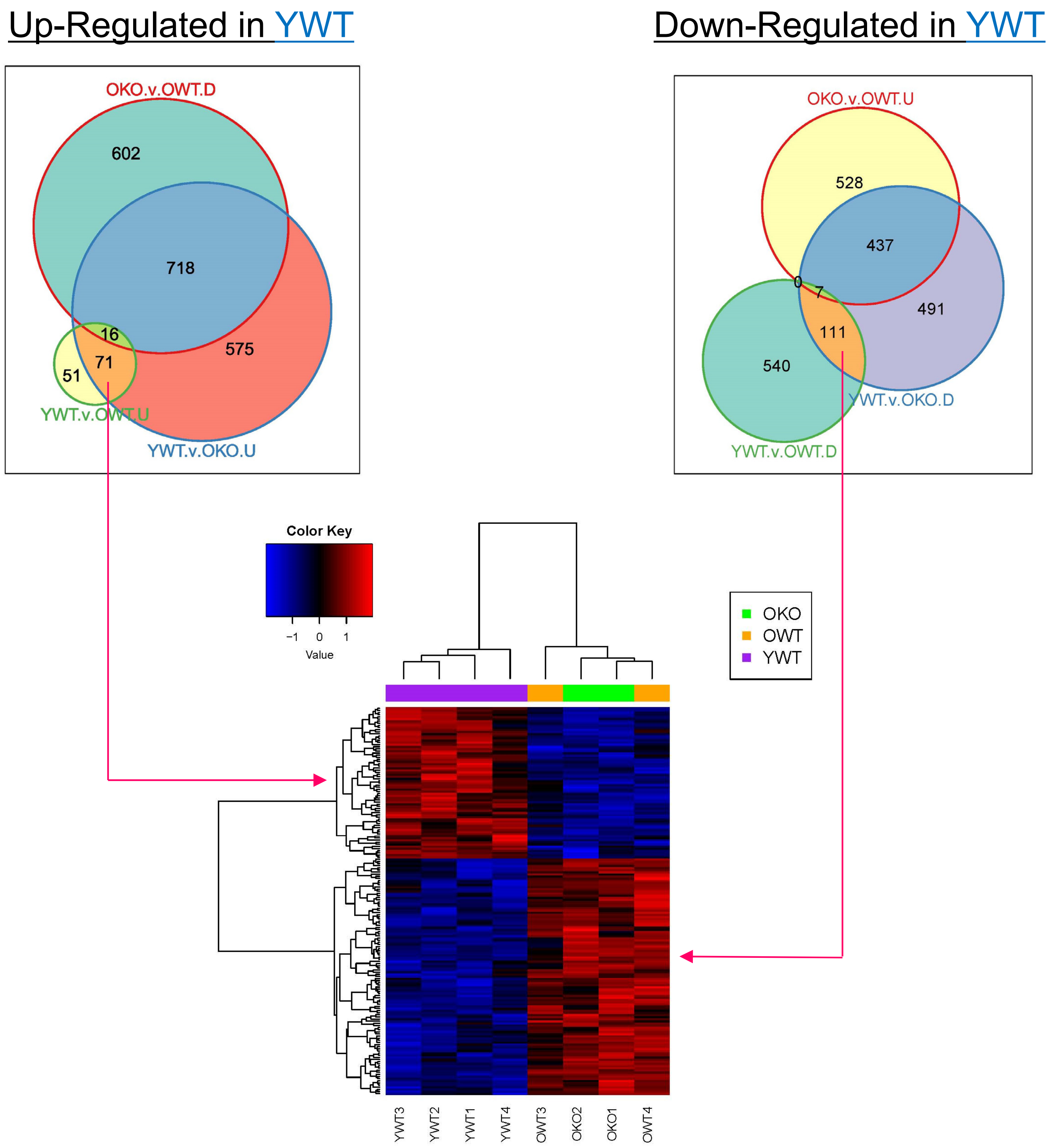

Supplementary Fig. 8 Identification of Sirt1-indepdent age-related gene changes. Venn diagram overlapping analysis identified 111 upregulated probesets in both OWT and OKO HSCs over YWT HSCs, and 71 downregulated probesets in both OWT and OKO HSCs over YWT HSCs. Additional overlapping with probesets that changed in OKO vs OWT only made a small difference for the results, confirming that the probesets identified were largely Sirt1-independent. The gene expression heatmap for the Sirt1-independent age-related genes in old mice were shown at the lower panel. The full list of these genes was provided as Supplementary Table 3.

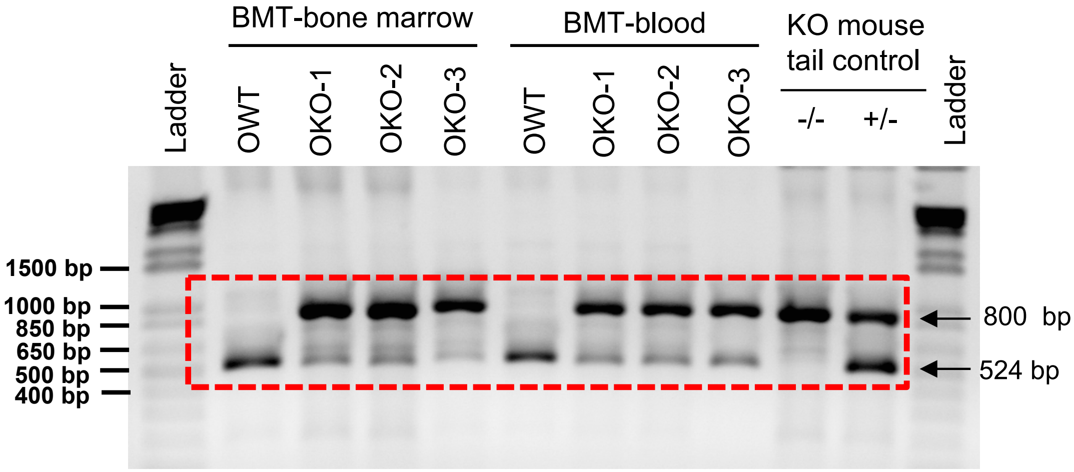

Supplementary Fig. 9 Uncropped gel image for Fig. 3f.

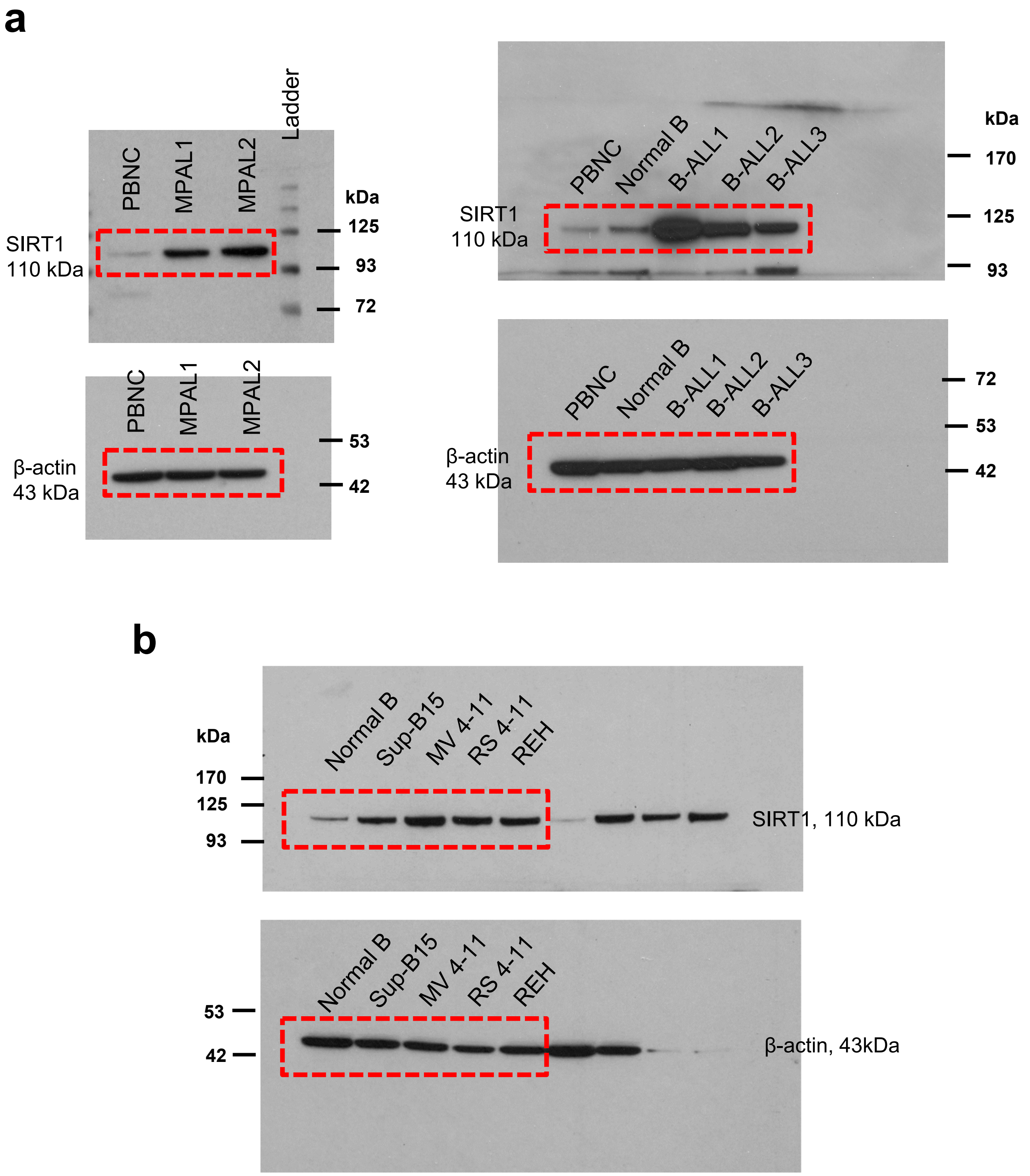

Supplementary Fig. 10 **a** Uncropped Western blot images for primary leukemia cells in Fig. 5a. **b** Uncropped Western blot images for leukemia cell lines in Fig. 5b.

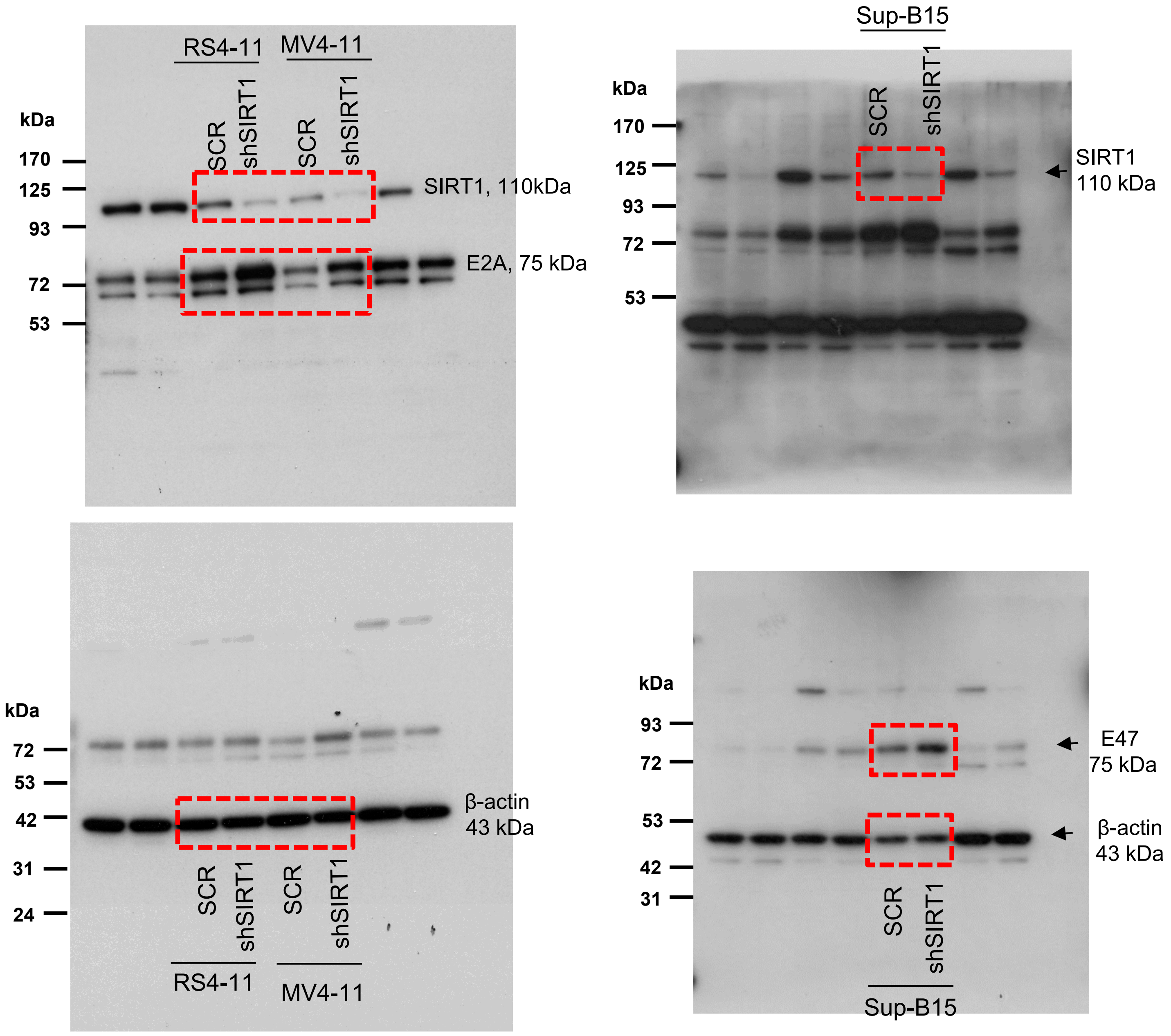

Supplementary Fig. 11 Uncropped Western blot images for Fig. 5d.

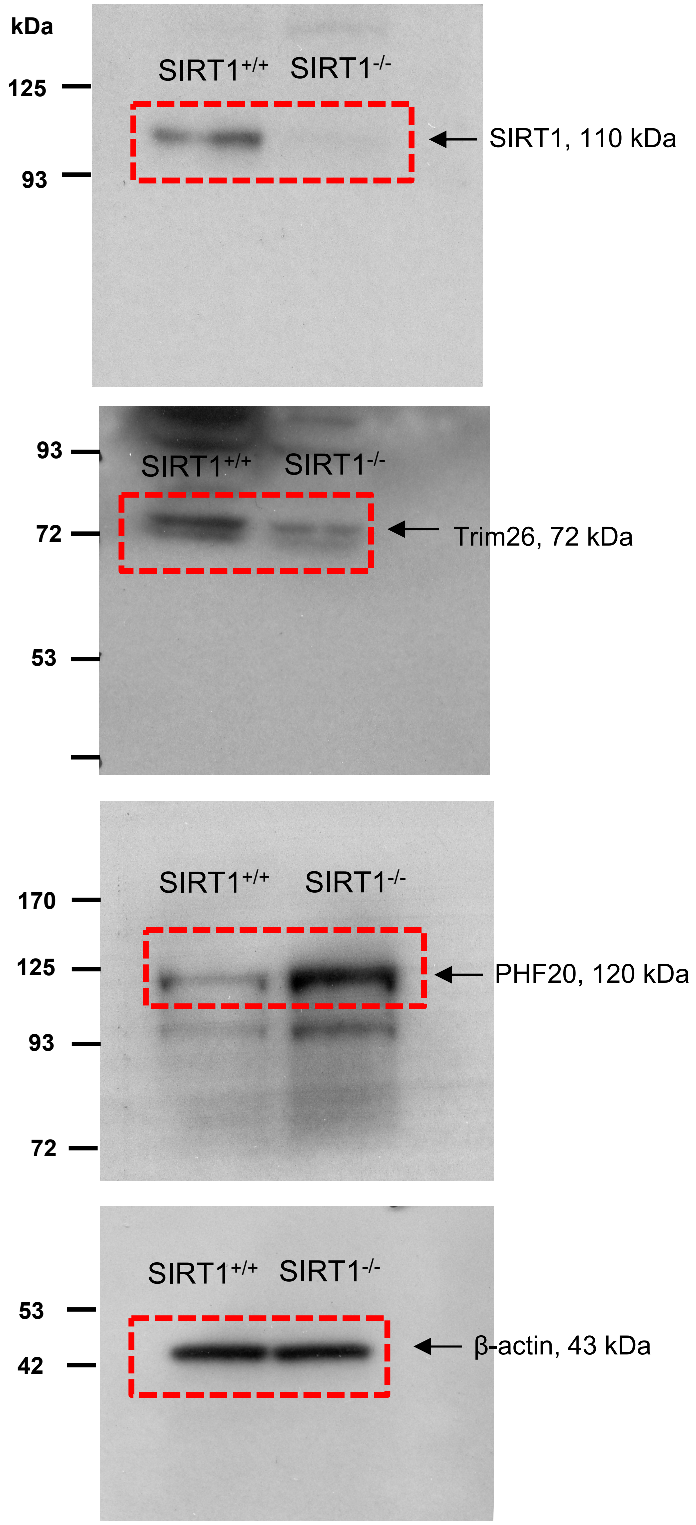

Supplementary Fig. 12 Uncropped Western blot images for Fig. 8e.

Supplementary Table 1

REAGENT or RESOURCE	SOURCE	IDENTIFIER
**Antibodies**		
Anti-mouse B220 (APC, PE)	BD Biosciences	(clone RA3-6B2) RRID: AB_398531
Anti-mouse PEcy7-CD3e	BD Biosciences	(clone 145-2c11) RRID: AB_394460
Anti-mouse PerCP5.5-Ter119	BD Biosciences	(clone Ter119) RRID: AB_10561844
Anti-mouse PE-Gr-1	BD Biosciences	(Clone RB6-8C5) RRID:AB_394644
Anti-mouse Mac-1	BD Biosciences	(clone M 1/70) RRID:AB_10561676
Anti-mouse APC-CD150	BioLegend	(clone TC15-12F12.2) RRID:AB_493461
Anti-mouse CD19	Biolegend	(clone 6D5) RRID:AB_830706
Anti-mouse CD43	eBioscience	(clone eBioR2/60) RRID:AB_465040
Anti-mouse CD127 (IL7a)	BD Biosciences	(Clone SB/199) RRID:AB_1727424
Anti-human E47	BD Biosciences	(Clone G127-32) RRID:AB_395228
Anti-mouse/human E2A	Santa Cruz Biotechnology	(Cat# sc-416) RRID:AB_627472
Anti-human β-Actin	Santa Cruz Biotechnology	(Cat# sc-47778 HRP) RRID:AB_271418
Anti-mouse SIRT1	Cell Signaling Technology	(Cat# 8469) RRID:AB_10999470
Anti-human SIRT1	Abcam	(Cat# ab32441) RRID:AB_777937
Anti-mouse Trim26	Santa Cruz Biotechnology	(Cat# sc-79774) RRID:AB_2256656
Anti-mouse PHF20	Cell Signaling Technology	(Cat# 3934) RRID:AB_2165078
Anti-mouse Ac-H4K16	Millipore	(polyclonal) Cat# 07-329, RRID:AB_310525
**Chemicals, medium, and supplements**		
Fetal bovine serum, defined, heat-inactivated	HyClone	SH30070.03IH
RPMI 1640 Medium with L-Glutamine and HEPES	Gibco	22400-089
GlutaMAX Supplement	ThermoFisher Scientific	35050061
2-Mercaptoethanol, 55 mM	ThermoFisher Scientific	21985023
MEM Non-Essential Amino Acids Solution (100X)	ThermoFisher Scientific	11140050
Phosphate-buffered saline	Irvine Scientific	9240
Penicillin-Streptomycin (100X)	Lonza	17602E
**Primer Sequence**		
**Gene name**	**Forward primer**	**Reverse primer**
mSirt1 (genotyping)	CTTGCACTTCAAGGGACCAA	R1: GTATACCCACCACATCTGAG<br>R2: CTACCACTCCTGGCTACCAA
mSirt1 (exon primers for RT-PCR)	ACACAGAGACGGCTGGAACT	AGACCTCCCAGACCCTCAAG
mTrim26	TGAAAGCCCTGGATTTTGTC	TGTTGTGTGTGATGTGCAGG
mDnmt1	AAGCCATCTCTTTCCAAGTCTTT	AGCTGTTCTGTCGTCTGCAA
mHOXA5	AGGTAGCGGTTGAAGTGGAA	CGCAAGCTGCACATTAGTCA
mMyc	TGAAGTTCACGTTGAGGGG	AGAGCTCCTCGAGCTGTTTG
mGsk3b	GTGGTTACCTTGCTGCCATC	GACCGAGAACCACCTCCTTT
mMyb	TGTCCTCAAAGCCTTTACCG	GTCCTCTGTCTTCCCACAGG
mEbf1	GGGTTTCCTGCATTCTTTAGG	CCGCTGTTGTGACAAGAAAA
mPax5	CAGCGTCGGTGCAGAGTAG	GGCCCACAGTCCTACCCTAT
mTcf3	AGTCCTGAGCCTGCAAACTG	GTGGGCTCTGACAAGGAACT
RPS28	CATCTGCAGTGGATGGTCGT	CCGTTGTAGCGGAAGGAGTT
RPS15	GCCCGTACTTCCTTTTCCGA	GGACATGTCGAGCAGTTGGT
PRL7a	GAAAGATGGGGGTGCCCTAC	GCACCCTTGTCTTCCGAGTT
RPL19	GTTAGGTGGGCAGCGGAG	GTTTGGCCAGCTTGACTGTG

RPL17	CTGTTGGGAGTGAGGTGCTT	ACATGGTCACGTGGTTTTGAAC	
RPS25	CGGAAAGTCGGCCAAAAAGG	ACTTGTCCCGAACTTTGCCT	
RPL26	TACATCGAACGAGTCCAGCG	ATAACGACCTTGCTGGGGTG	
RPL18	GGTTCCGTCTTTCCGGACC	GGTCCTTGTTGTGGCGAATG	
RPL12	GGCAACGGTGCAACTTTCTT	GACTTTGACCTCGTTGGGGT	
hTCF3	CCTGTCACCAACGGGAAG	AAGGAGGAGCTGCTCTGGTC	
hEBF	GTGGCAACCGAAATGAGACT	GGTTTCCCGCATTCTTTAGG	
hPAX5	CATCTTCACCACCACAGAGC	AGATTGGCCTTCATGTCGTC	
hTrim26	GTCAGGAATGGAGAGAAGGGT	CTGCTATGGTTCTGCCCTTT	
DH3-1 (V(D)J assay)	AGGCTCTGAGATCCCTAGACAG		
DH-5-1 (V(D)J assay)	ACAAGCTTCAAAGCACAATGCC TGGCT		
DH3-2 (V(D)J assay)	GGGTCTAGACTCTCAGCCGGCTC CCTCAGGG		
DH5-2 (V(D)J assay)	ACGTCGACTTTTSTCAAGGGATC TACTACTGT		
GL-3-2 (V(D)J assay)	CGAAGTACCAGTAGCAC		
GL-5-1 (V(D)J assay)	CCCGGACAGAGCAGGCAGGTGG		
GL-5-2 (V(D)J assay)	GAGTTGACTGAGAGGACAG		
**Plasmids**			
pSico_PGK puro	Addgene plasmid # 12084; gift from Tyler Jacks	RRID:Addgene_12084	
pSico_puro_shhSIRT1	Yuan. H. et.al Blood, 2012		
pSico_puro_shmSirt1	Yuan. H. et.al Blood, 2012		
Critical Commercial Assays			
PicoPure RNA Isolation Kit	ThermoFisher Scientific	KIT0204	
Direct-zol RNA Microprep	Zymo Research	R2061	
EasySep mouse lineage Selection Kit II	Stem Cell Technologies	Cat# 17855	
Fixation/Permeabilization Solution Kit	BD Biosciences	Cat# 554714	
**Experimental model: cell lines**			
Sup B-15	ATCC	CRL-1929	
MV4-11	ATCC	CRL-9591	
RS4-11	ATCC	CRL-1873	
REH	ATCC	CRL-8286	
**Experimental model: organisms**			
Mouse: Balb/c	Taconic		
**Software and Algorithms**			
GraphPad Prism GraphPad Software	GraphPad Software	https://www.graphpad.com/	
Flowjo	Tree Star Inc.	https://www.flowjo.com/solutions/flowjo	
Zen	Zeiss	https://www.zeiss.com/microscopy/int/products/microscope-software/zen.html	

Partek<br>(Partek 6.6 beta, release 6.12.0530).	Partek Inc	https://www.partek.com/	
GSEA (v.2.07).<br>(Gene Set Enrichment Analysis)	Broad Institute	https://www.gsea-msigdb.org/gsea/index.jsp	
MSigDB (v3.0)<br>(Molecular Signatures DataBase)	Broad Institute	http://www.gsea-msigdb.org/gsea/msigdb/index.jsp<br>(C2 Canonical Pathway gene sets)	
