# Supplementary material for: Loss of SIRT1 inhibits hematopoietic stem cell aging and age-dependent mixed phenotype acute leukemia
Source: Commun Biol. 2022 Apr 28;5:396. doi: 10.1038/s42003-022-03340-w (PMC9051098; doi:10.1038/s42003-022-03340-w)
Supplement: Supplementary file 3 — Description of Additional Supplementary Files [file 42003_2022_3340_MOESM3_ESM.pdf]

Description of Additional Supplementary Files

File name: Supplementary Data 1
Description: GSEA report for enriched pathways in OWT in the analysis OWT vs YWT.

File name: Supplementary Data 2
Description: GSEA report for enriched pathways in OWT in the analysis of OKO vs OWT.

File name: Supplementary Data 3
Description: SIRT1-independent aging gene list.

File name: Supplementary Data 4
Description: SIRT1-independent aging gene enrichment

File name: Supplementary Data 5
Description: Sirt1-regulated genes identified by Venn diagram analysis

File name: Supplementary Data 6
Description: Extra Gene Sets for MSigDB
