# Supplementary material for: Loss of SIRT1 inhibits hematopoietic stem cell aging and age-dependent mixed phenotype acute leukemia
Source: Commun Biol. 2022 Apr 28;5:396. doi: 10.1038/s42003-022-03340-w (PMC9051098; doi:10.1038/s42003-022-03340-w)
Supplement: Supplementary file 10 — Reporting Summary [file 42003_2022_3340_MOESM10_ESM.pdf]

nature portfolio

Corresponding author(s): WenYong Chen

Last updated by author(s): Aug 9, 2021

Reporting Summary

Nature Portfolio wishes to improve the reproducibility of the work that we publish. This form provides structure for consistency and transparency in reporting. For further information on Nature Portfolio policies, see our Editorial Policies and the Editorial Policy Checklist.

Statistics

For all statistical analyses, confirm that the following items are present in the figure legend, table legend, main text, or Methods section.

n/a	Confirmed	
☐	☒	The exact sample size (*n*) for each experimental group/condition, given as a discrete number and unit of measurement
☐	☒	A statement on whether measurements were taken from distinct samples or whether the same sample was measured repeatedly
☐	☒	The statistical test(s) used AND whether they are one- or two-sided<br>*Only common tests should be described solely by name; describe more complex techniques in the Methods section.*
☒	☐	A description of all covariates tested
☒	☐	A description of any assumptions or corrections, such as tests of normality and adjustment for multiple comparisons
☐	☒	A full description of the statistical parameters including central tendency (e.g. means) or other basic estimates (e.g. regression coefficient) AND variation (e.g. standard deviation) or associated estimates of uncertainty (e.g. confidence intervals)
☒	☐	For null hypothesis testing, the test statistic (e.g. *F*, *t*, *r*) with confidence intervals, effect sizes, degrees of freedom and *P* value noted<br>*Give P values as exact values whenever suitable.*
☒	☐	For Bayesian analysis, information on the choice of priors and Markov chain Monte Carlo settings
☒	☐	For hierarchical and complex designs, identification of the appropriate level for tests and full reporting of outcomes
☒	☐	Estimates of effect sizes (e.g. Cohen's *d*, Pearson's *r*), indicating how they were calculated

Our web collection on statistics for biologists contains articles on many of the points above.

Software and code

Policy information about availability of computer code

Data collection	We used open source software for microarray data collection and analysis including Partek® Genomics Suite® (Partek Inc. release 6.12.0530), Gene Set Enrichment Analysis software (GSEA, v2.07) and Molecular Signature Database (MSigDB; v3.0). Graphpad Prizm 8.0 was used for statistical analysis and graphing for other data.
Data analysis	We used open source software for microarray data collection and analysis including Partek® Genomics Suite® (Partek Inc. release 6.12.0530), Gene Set Enrichment Analysis software (GSEA, v2.07) and Molecular Signature Database (MSigDB; v3.0). Graphpad Prizm 8.0 was used for statistical analysis and graphing for other data.

For manuscripts utilizing custom algorithms or software that are central to the research but not yet described in published literature, software must be made available to editors and reviewers. We strongly encourage code deposition in a community repository (e.g. GitHub). See the Nature Portfolio guidelines for submitting code & software for further information.

Data

Policy information about availability of data

All manuscripts must include a data availability statement. This statement should provide the following information, where applicable:

- Accession codes, unique identifiers, or web links for publicly available datasets
- A description of any restrictions on data availability
- For clinical datasets or third party data, please ensure that the statement adheres to our policy

The data generation and analysis are described in the Methods section. GSEA reports of microarray data are available as Table S1 and Table S2, and the list of genes identified by Venn diagram analysis is provided in Table S3. The GEO accession number for the raw microarray data reported in this paper is GSE169387. These data will be publicly available without restriction.

Field-specific reporting

Please select the one below that is the best fit for your research. If you are not sure, read the appropriate sections before making your selection.

☒ Life sciences ☐ Behavioural & social sciences ☐ Ecological, evolutionary & environmental sciences

For a reference copy of the document with all sections, see nature.com/documents/nr-reporting-summary-flat.pdf

Life sciences study design

All studies must disclose on these points even when the disclosure is negative.

Sample size	The mouse group size was determined according to Dell RB, et al. ILAR J 2002, 43: 207-213. In a typical setting, we assumed a 99% power to detect phenotypic difference in as low as 40% of animals, which required about 10 mice per group. Overall, we assigned 8 to 12 mice per group. The adjustment of group size occurred depending on the availability of mice at the time of experiment and/or the phenotypic strength.
Data exclusions	no data excluded
Replication	Key biological experiments were replicated as described in the manuscript.
Randomization	Lethally irradiated recipient mice were randomly distributed among study groups.
Blinding	The group allocation was not blinded to the investigators as we didn't have enough personnel to carry out the double-blinded study.

Reporting for specific materials, systems and methods

We require information from authors about some types of materials, experimental systems and methods used in many studies. Here, indicate whether each material, system or method listed is relevant to your study. If you are not sure if a list item applies to your research, read the appropriate section before selecting a response.

Materials & experimental systems

n/a	Involved in the study	
☐	☒	Antibodies
☐	☒	Eukaryotic cell lines
☒	☐	Palaeontology and archaeology
☐	☒	Animals and other organisms
☒	☐	Human research participants
☒	☐	Clinical data
☒	☐	Dual use research of concern

Methods

n/a	Involved in the study	
☒	☐	ChIP-seq
☐	☒	Flow cytometry
☒	☐	MRI-based neuroimaging

Antibodies

Antibodies used

Anti-mouse B220 (APC, PE) BD Biosciences (clone RA3-6B2) RRID: AB_398531
Anti-mouse PEcy7-CD3e BD Biosciences (clone 145-2c11) RRID: AB_394460
Anti-mouse PerCP5.5-Ter119 BD Biosciences (clone Ter119) RRID: AB_10561844
Anti-mouse PE-Gr-1 BD Biosciences (Clone RB6-8C5) RRID:AB_394644
Anti-mouse Mac-1 BD Biosciences (clone M 1/70) RRID:AB_10561676
Anti-mouse APC-CD150 BioLegend (clone TC15-12F12.2) RRID:AB_493461
Anti-mouse CD19 Biolegend (clone 6D5) RRID:AB_830706
Anti-mouse CD43 eBioscience
(clone eBioR2/60) RRID:AB_465040
Anti-mouse CD127 (IL7a) BD Biosciences (Clone SB/199) RRID:AB_1727424
Anti-human E47 BD Biosciences (Clone G127-32) RRID:AB_395228
Anti-mouse/human E2A Santa Cruz Biotechnology (Cat# sc-416) RRID:AB_627472
Anti-human β-Actin Santa Cruz Biotechnology (Cat# sc-47778 HRP) RRID:AB_271418
Anti-mouse SIRT1 Cell Signaling Technology (Cat# 8469) RRID:AB_10999470
Anti-human SIRT1 Abcam (Cat# ab32441) RRID:AB_777937
Anti-mouse Trim26 Santa Cruz Biotechnology (Cat# sc-79774) RRID:AB_2256656
Anti-mouse PHF20 Cell Signaling Technology (Cat# 3934) RRID:AB_2165078
Anti-mouse Ac-H4K16 Millipore (polyclonal) Cat# 07-329, RRID:AB_310525

Validation

These antibodies have been validated in the vendors' web sites and well cited by numerous publications from our and others' previous studies (some were cited in the manuscript).

Eukaryotic cell lines

Policy information about cell lines

Cell line source(s)	ATCC
Authentication	Four cell lines (Sup B-15, MV4-11, RS4-11 and REH) were directly ordered from ATCC and were not separately authenticated during the study.
Mycoplasma contamination	all cell lines were tested negative for mycoplasma contamination.
Commonly misidentified lines (See ICLAC register)	None

Animals and other organisms

Policy information about studies involving animals; ARRIVE guidelines recommended for reporting animal research

Laboratory animals	We used wild type BALB/c mice and Sirt1 KO mice backcrossed to BALB/c in the study. Mice were aged for the studies as detailed in the manuscript, and both sexes were used.
Wild animals	N/A
Field-collected samples	N/A
Ethics oversight	All animal experiments were conducted under a protocol approved by the City of Hope Institutional Animal Care and Use Committee.

Note that full information on the approval of the study protocol must also be provided in the manuscript.

Flow Cytometry

Plots

Confirm that:

- [x] The axis labels state the marker and fluorochrome used (e.g. CD4-FITC).
- [x] The axis scales are clearly visible. Include numbers along axes only for bottom left plot of group (a 'group' is an analysis of identical markers).
- [x] All plots are contour plots with outliers or pseudocolor plots.
- [x] A numerical value for number of cells or percentage (with statistics) is provided.

Methodology

Sample preparation	Blood and bone marrow samples were harvested from mice, processed and then stained with antibodies as detailed in the methods.
Instrument	BD LSR Fortessa cell analyzer
Software	FlowJo (v10.6.1).
Cell population abundance	The abundance of cell population varied largely depending on the cell fractions to be studied as detailed in the manuscript. The purity of SP fractions was determined by post-sort flow analysis to be higher than 97% (Wang Z. et al Stem Cell 2015;33:3437–3451)
Gating strategy	We gated most live mononucleated cells on the starting SSC/FSC plots. The boundary for "positive" and "negative" staining fractions was defined by the clear gap between two. More detailed gating strategies have been shown previously in Wang Z. et al Stem Cell 2015;33:3437–3451.

- [ ] Tick this box to confirm that a figure exemplifying the gating strategy is provided in the Supplementary Information.
